# Transfer of Enteric Viruses Adenovirus and Coxsackievirus and Bacteriophage MS2 from Liquid to Human Skin

**DOI:** 10.1128/AEM.01809-18

**Published:** 2018-10-30

**Authors:** Ana K. Pitol, Heather N. Bischel, Alexandria B. Boehm, Tamar Kohn, Timothy R. Julian

**Affiliations:** a Eawag, Swiss Federal Institute of Aquatic Science and Technology, Dübendorf, Switzerland; b Laboratory of Environmental Chemistry, School of Architecture, Civil, and Environmental Engineering (ENAC), École Polytechnique Fédérale de Lausanne (EPFL), Lausanne, Switzerland; c Department of Civil & Environmental Engineering, University of California, Davis, Davis, California, USA; d Department of Civil and Environmental Engineering, Stanford University, Stanford, California, USA; e Swiss Tropical and Public Health Institute, Basel, Switzerland; f University of Basel, Basel, Switzerland; Centers for Disease Control and Prevention

**Keywords:** adenovirus, coxsackievirus, enteric virus, MS2, virus transfer

## Abstract

Indirect exposure to waterborne viruses increases the risk of infection, especially among children with frequent hand-to-mouth contacts. Here, we quantified the transfer of one bacteriophage (MS2) and two enteric viruses (adenovirus and coxsackievirus) from liquid to skin. MS2, a commonly used enteric virus surrogate, was used to compare virus transfer rates in a volunteer trial to those obtained using human cadaver skin and synthetic skin. MS2 transfer to volunteer skin was similar to transfer to cadaver skin but significantly different from transfer to synthetic skin. The transfer of MS2, adenovirus, and coxsackievirus to cadaver skin was modeled using measurements for viruses attaching to the skin (adsorbed) and viruses in liquid residual on skin (unadsorbed). We find virus transfer per surface area is a function of the concentration of virus in the liquid and the film thickness of liquid retained on the skin and is estimable using a linear model. Notably, the amount of MS2 adsorbed on the skin was on average 5 times higher than the amount of adenovirus and 4 times higher than the amount of coxsackievirus. Quantification of pathogenic virus retention to skin would thus be overestimated using MS2 adsorption data. This study provides models of virus transfer useful for risk assessments of water-related activities, demonstrates significant differences in the transfer of pathogenic virus and MS2, and suggests cadaver skin as an alternative testing system for studying interactions between viruses and skin.

**IMPORTANCE** Enteric viruses (viruses that infect the gastrointestinal tract) are responsible for most water-transmitted diseases. They are shed in high concentrations in the feces of infected individuals, persist for an extended period of time in water, and are highly infective. Exposure to contaminated water directly (through ingestion) or indirectly (for example, through hand-water contacts followed by hand-to-mouth contacts) increases the risk of virus transmission. The work described herein provides a quantitative model for estimating human-pathogenic virus retention on skin following contact with contaminated water. The work will be important in refining the contribution of indirect transmission of virus to risks associated with water-related activities.

## Abstract (precis)

Enteric viruses (viruses that infect the gastrointestinal tract) are responsible for most water-transmitted diseases. They are shed in high concentrations in the feces of infected individuals, persist for an extended period of time in water, and are highly infective. Exposure to contaminated water directly (through ingestion) or indirectly (for example, through hand-water contacts followed by hand-to-mouth contacts) increases the risk of virus transmission. The work described herein provides a quantitative model for estimating human-pathogenic virus retention on skin following contact with contaminated water. The work will be important in refining the contribution of indirect transmission of virus to risks associated with water-related activities.

## INTRODUCTION

Waterborne viruses, including noroviruses, adenovirus, enteroviruses, astrovirus, rotavirus, and hepatitis A virus, are responsible for a significant proportion of recreational and drinking water outbreaks (1–3). Waterborne viruses are typically introduced to water sources through fecal contamination (1). Once in the water, viruses can be transmitted to people either directly (through ingestion) or indirectly (i.e., through hand-water or hand-fomite contacts followed by hand-to-mouth contacts). Indirect transmission is likely a substantial contributor to infection risks from water contacts, especially in scenarios involving children (4–6), among whom hand-to-mouth contacts are frequent (7).

The importance of indirect transmission of pathogens via contaminated fomites has been explored using microbial risk assessments (8–13). Since risk assessments of fomite-mediated transfer evaluate the risk from indirect transmission, it is not surprising that there is extensive literature quantifying pathogen transfer between hands and fomites (14–22). In contrast, risk assessments of recreational and occupational water exposure focus almost exclusively on the direct ingestion of contaminated liquid (23–30). Only a few risk assessments of waterborne pathogens consider indirect transmission due to hand-to-mouth contacts (31–33), and data on the transfer of viruses between hands and liquids are sparse (34).

To account for indirect transmission of waterborne pathogens, estimates of the number of viruses transferred between liquid and skin are needed. Prior work on fomite-mediated transfer suggests that transfer can be described as a percentage, where the fraction of pathogens transferred to the recipient surface (i.e., skin) is a proportion of the total number of pathogens on the donor surface (i.e., fomite) prior to contact (18–20). In contrast, liquid-mediated transfer requires accounting for both the pathogens that transfer to the recipient surface (i.e., skin) from the donor matrix (i.e., water) through retention of liquid residual on the skin, as well as pathogens that adsorb to the skin surface (32). Our prior work investigating bacteriophage suggests that the quantity of bacteriophage transferred from liquid to skin can be estimated as the sum of the virus adsorbed on the skin plus the virus in the liquid residual on the skin. The magnitude of virus adsorption to skin is a function of the concentration of virus in the liquid matrix. The liquid characteristics of pH (6, 7.5, and 9) and ionic strength (10 mM and 550 mM) were previously shown to have no influence on the adsorption of bacteriophage to the skin (34). Additionally, our prior studies suggest that bacteriophage type has a small but significant influence on virus adsorption, as also observed in fomite-mediated transfer studies (15, 20). Conversely, virus retained in the residual liquid on the skin is only dependent on virus concentration in the liquid matrix (34).

The transfer of pathogenic viruses between the environment (e.g., water and fomites) and human skin is typically approximated using bacteriophages as pathogenic virus surrogates (18–20, 34). Bacteriophages are commonly used as human-virus surrogates because they have similar shapes, sizes, morphologies, and isoelectric points as human-pathogenic viruses (35, 36). Additionally, bacteriophages are safe to use, enabling studies with human volunteers (37). Bacteriophages are also relatively easy to enumerate, with rapid low-cost methods for quantification of infectivity (37). Nevertheless, bacteriophages do not always mimic the behavior of human viruses. For example, bacteriophages MS2 and ΦX174 failed to emulate the adsorption behavior of different enteric viruses tested on inanimate objects, such as sand and membrane filters (38, 39). Additionally, the same viral surrogate can describe the pathogen adequately in some scenarios and not in others based on the environmental conditions (e.g., liquid pH and ionic strength) (40, 41). Since the environment influences the extent to which the surrogate mimics the behavior of the pathogen of interest (42), the use of pathogen surrogates should be validated in the specific scenario under study (41, 43).

The use of pathogenic viruses in studies of virus-skin interactions involves risks to human volunteers, necessitating the consideration of safer skin surrogates. The majority of research on the interaction between human pathogens and the skin (e.g., studies of pathogen transfer, survival, and susceptibility to biocides) has been performed using the hands (17, 44) and fingers (15, 16, 18, 19, 34, 44–46) of volunteers. These studies rely on the use of pathogens or surrogates that do not pose a significant risk to the subjects. However, to understand the interaction of virulent pathogens with human skin and to assess the validity of pathogen surrogates for these organisms, alternatives to human volunteer skin are essential. Animal skin (45–47), synthetic skin (48), and fragments of human skin obtained from surgery (49–51) and from cadavers (52) have been used to study microbial interactions with skin while preventing unnecessary risks to volunteers. The use of volunteer skin surrogates necessitates validation through a comparison of the study outcome obtained using surrogates to that obtained using volunteer skin.

This study informs our understanding of the indirect transmission of enteric viruses through contact with liquids. Specifically, the study quantifies and models the transfer of two pathogenic viruses (human adenovirus type 2 and human coxsackievirus B5) from liquid to skin and compares the results to the transfer of bacteriophage MS2, a commonly used enteric virus surrogate (see Table S1 in the supplemental material). Because the use of pathogenic viruses poses risks to study volunteers, we furthermore examined skin surrogates (cadaver and synthetic skin) that could be used to study pathogens directly, providing a better understanding of virus transfer from liquids to human skin.

## RESULTS

### MS2 transfer to different skin models.

**FIG 1:**
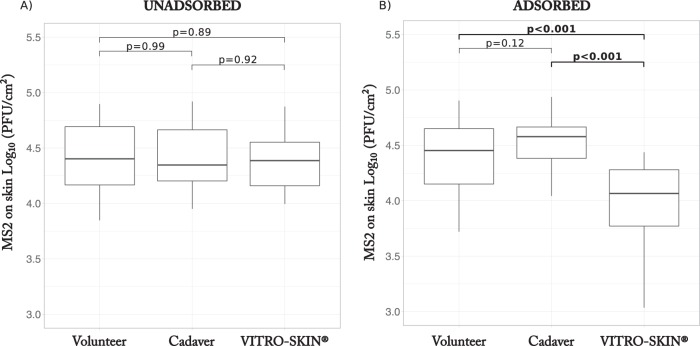
Bacteriophage MS2 (log_10_ transformed virus density) transferred to the skin, unadsorbed and adsorbed, as a function of skin type. The boxplots summarize the number of MS2 bacteriophages unadsorbed (A) and adsorbed (B) on three different skin types (volunteer skin, cadaver skin, and Vitro-Skin). The transfer studies were conducted using liquid containing 10^7.5^ PFU/ml bacteriophage MS2. The boxplots summarize the data from 28 individual transfers for each skin type. The top and bottom of the boxplots represent the 25th and 75th percentiles, the center line represents the median value, and the whiskers extend to the highest and lowest concentrations. The statistical significance of the difference between each pair of skin types is indicated above the boxplots by the corresponding *P* values, with those <0.001 in bold.

A total of 84 individual transfer events were performed using MS2 bacteriophage and three different skin types, as follows: the skin of healthy volunteers, the skin of cadavers, and the synthetic skin Vitro-Skin N-19 (Table 1, experiment A). In a comparison of virus transfer from liquid to the three different skin types, we found no significant effect of the skin used on the number of bacteriophage MS2 unadsorbed on the skin [analysis of variance (ANOVA), *F* (2, 79) = 0.12, *P* = 0.88] (Fig. 1A). However, we did find a significant influence of the skin type on the amount of MS2 adsorbed on the skin [ANOVA, *F* (2, 79) = 24.55, *P* < 0.001, *η_p_*^2^ (partial eta squared) = 0.383] (Fig. 1B). Specifically, the adsorption of MS2 to Vitro-Skin was 0.4 log_10_ less than that to volunteer skin (Tukey's *post hoc* comparison, *P* < 0.001), whereas the adsorption of MS2 to volunteer skin was not significantly different from the adsorption to cadaver skin (*P* = 0.12). None of the volunteers, skin specimens, and synthetic skins were contaminated with male-specific (F^+^) coliphage prior to the experiments, as shown by the absence of plaques in all negative controls.

**TABLE 1:** Description of experiments in this study in comparison to prior work by Pitol et al.^*a*^

Expt by study	Skin type (body part)	Virus	No. of specimens/volunteers	No. of times a set of expts was performed	No. of transfer events^*b*^
This study					
A	Cadaver (hand/arm)	MS2	3	4	28
	Vitro-Skin	MS2	4	4	28
	Volunteer (hand/arm)	MS2	4	4	28
B	Cadaver (hand/arm)	MS2	5	8	48
		Adenovirus	5	8	45
		Coxsackievirus	5	8	47
Pitol et al. (34)	Volunteer (hand)	MS2	7	7	70

a All experiments were performed at a seeding concentration of 10^6^ to 10^8^ PFU or MPN/ml. MPN was calculated using five replicates per concentration (i.e., 0 to 5 positive results).

b Equivalent to the sample size or the total number of replicates.

### Comparison of different experimental approaches to measure virus transfer (finger dipping versus droplet).

A subset of the experimental data obtained from experiment B (Table 1), which consisted of 48 liquid-to-skin transfer events performed using bacteriophage MS2, was used to compare the results of this study (cadavers and volunteers) with the results of our previous study (volunteers). The transfer method of our previous study (34) consisted of dipping the finger directly into liquid with bacteriophages (Fig. S2). In contrast, the transfer method in this study consisted of applying and removing a droplet of liquid with virus on the skin using a pipette (Fig. S1 and S2).

**FIG 2:**
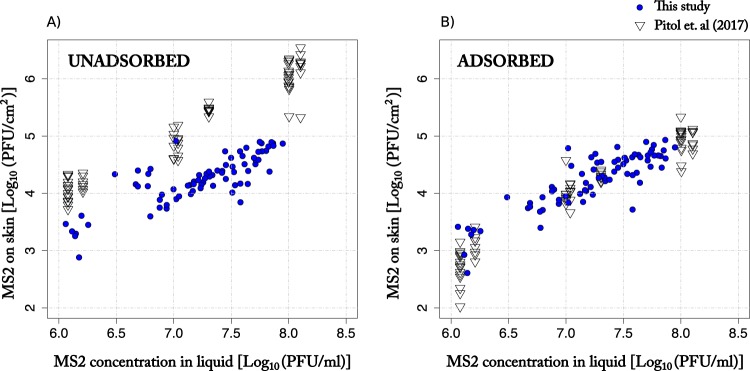
Influence of experimental method on virus transfer to skin (adsorbed and unadsorbed). The plots show the number of MS2 (log_10_ transformed) transferred to the skin per surface area as a function of the concentration of MS2 (log_10_ transformed) in the liquid. Virus transfer was measured using two different experimental methods, as follows: (i) the finger-dipping method (data from Pitol et al. [34]), and (ii) the droplet method (this study). In the finger-dipping method, a set of 7 volunteers were asked to dip their fingers into a glass containing a saline solution with MS2, and the skin was sampled afterward. Each transfer event is represented by an open triangle. In the droplet method, the transfer events were carried out in cadavers' and volunteers' hands or arms by applying and removing a 20-μl droplet of buffer containing MS2. Each transfer event is represented by a blue circle.

Both studies showed a linear relationship between the log_10_-transformed number of viruses transferred to the skin per surface area (adsorbed and unadsorbed) and the log_10_-transformed concentration of virus in the liquid applied to the skin (Fig. 2). The numbers of bacteriophage MS2 adsorbed on the skin per surface area (in PFU per square centimeter) were similar in the two studies (Fig. 2B). In contrast, the amount of unadsorbed MS2 retained on the skin was on average eight times lower (∼1 log_10_ PFU/cm^2^) for the droplet method used in this study than for the finger-dipping method (34) (Fig. 2A). The findings suggest the experimental method influences the volume of liquid retained on the skin after contact and therefore also the number of viruses retained. Assuming that the unadsorbed virus retained in the liquid on the skin per surface area *n* (virus/cm^2^) can be estimated by multiplying the concentration of virus in the liquid (*C* [virus/cm^3^]) by the liquid film thickness (*h* [cm]), *n* = *C h* (34), then the film thickness of the liquid retained on the skin was (mean ± standard deviation) 1.4 × 10^−3^ cm ± 1.3 × 10^−3^ cm for the droplet method (this study) and 1.1 × 10^−4^ cm ± 5.2 × 10^−3^ cm for the finger-dipping method.

### Transfer of pathogenic viruses and bacteriophages.

A total of 140 virus transfer events from liquid to cadaver hands and/or arms were evaluated, with 48 events using bacteriophage MS2, 45 events using adenovirus, and 47 events using coxsackievirus (Table 1, experiment B). As expected, the number of bacteriophage MS2 and viral pathogens (adenovirus or coxsackievirus) remaining on the skin per surface area due to residual liquid transfer (the unadsorbed fraction) was proportional to *C*, the seeding concentration of virus in the liquid (Fig. S3). Since the number of unadsorbed virus retained on the skin per surface area is influenced by the experimental method (Fig. 2A) and can be estimated as *n* = *C h*, as described previously (34), we proceeded with analyzing only the adsorbed fraction. Further analysis of the unadsorbed fraction can be found in the supplemental material (Table S2). Multiple regression was used to predict the log_10_-transformed number of viruses adsorbed on cadaver skin per surface area as a function of virus seeding concentration, virus species, specimen used, and body part [*F*(8, 131) = 40.42, *P* < 0.001, *R^2^* = 0.69] (Table 2, model 1). Specifically, the number of viruses per surface area adsorbed on the skin was significantly associated with the seeding concentration of virus in the liquid, virus species, and specimen but not body part. Both adenovirus and coxsackievirus concentrations per surface area were significantly different than that of MS2 when controlling for all the factors (Table 2, model 1; *P* < 0.001). No significant difference in adsorption was observed between adenovirus and coxsackievirus, as shown by multiple regression analysis using adenovirus as the reference pathogen (Table S3, *P* = 0.18). Additionally, cadaver specimen 3 was significantly different from specimens 1 (*P* = 0.007), 2 (*P* = 0.009), and 4 (*P* = 0.011) but not different from specimen 5 (*P* = 0.102) (Table S3). No significant difference was observed in a comparison of the density of viruses adsorbed on the hand compared to that on the arm (*P* = 0.89) (Table 2, model 1).

**TABLE 2:**
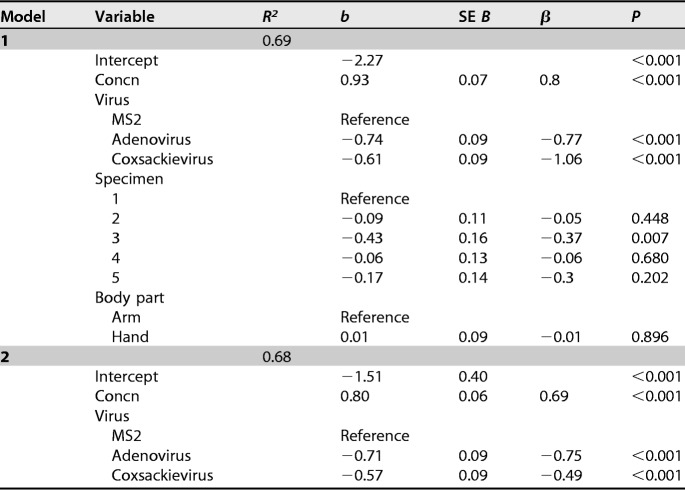
Multiple regression analysis for the log_10_-transformed number of viruses adsorbed on the skin per surface area as a function of the log_10_-transformed concentration of virus in the liquid, virus type, specimen used, and body part^*a*^

a Two models are presented. Model 1 includes all the predictor variables, and model 2 includes the predictor variables that are statistically significant and relevant for modeling virus transfer. The table shows the goodness of fit of the regression models (*R^2^*), the coefficient of the regression (*b*), standard error (SE *B*), standardized regression coefficient (β), and significance level (*P*) of virus adsorbed on the skin. “Reference” refers to the reference group used in the multiple regression analysis.

### Virus transfer models.

Results from multiple regression (Table 2, model 2) can be used as a predictive model to estimate the adsorption of virus as a function of concentration of viruses in the liquid and virus type. For the predictive model, both body part and specimen were removed (Table 2, model 2). Body part was not significant in the model and, although specimen was significant, it is impractical to include interspecimen variation in the predictive models. Furthermore, the inclusion of specimen minimally improves model explanatory power, as shown by the *R*^2^ values of model 1 compared to model 2 (Table 2). Therefore, we suggest using the following equation to model virus adsorption to the skin:

(1) $$\log_{10}\;n=m\;\log_{10}C\;+\;b_{0}\;+\;b_{virus}$$

which can be transformed to

(2) $$n=10^{\left(b_{0}\;+\;b_{virus}\right)}\;C^{m}$$

where *n* (viruses/cm^2^) is the number of viruses on the skin per surface area, *m* and *b*_0_ are the slope and intercept of the empirically derived regression, respectively, *C* (viruses/cm^3^) is the concentration of virus in the liquid, and *b*_virus_ is the virus coefficient, which quantifies the deviation of the model attributable to virus type (MS2, adenovirus, or coxsackievirus). Because MS2 is the reference virus, *b*_virus_ for MS2 is 0.

**FIG 3:**
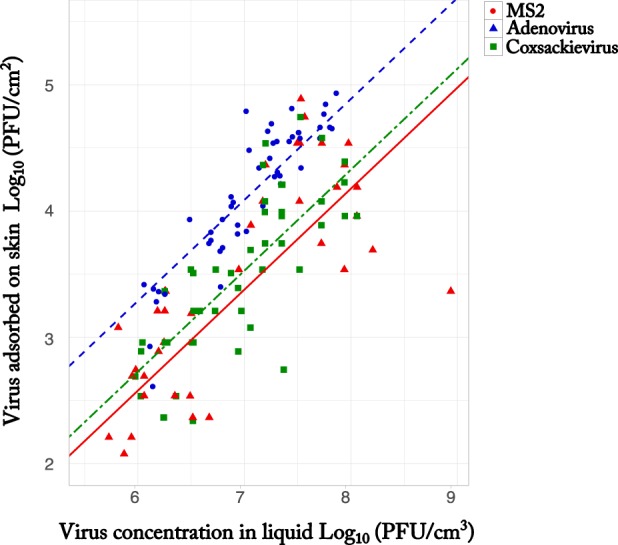
Number of bacteriophages and pathogenic viruses adsorbed on the skin per surface area as a function of seeding concentration. The plots show the log_10_-transformed MS2 (red circles), adenovirus (blue triangles), and coxsackievirus (green squares) adsorbed on the skin per surface area as a function of the log_10_-transformed concentration of virus in the liquid. The regression lines represent the multiple regression models for the number of viruses adsorbed per surface area as a function of concentration.

Since the total number of viruses transferred from liquid to skin can be described as the number of viruses adsorbed on the skin per surface area plus the number of unadsorbed viruses on the skin per surface area, we can therefore estimate virus transfer using the following equations (Fig. 3):

(3) $$n\left(\frac{\text{MS}2}{{\text{cm}}^{2}}\right)=10^{-1.5}\;C^{0.8}\;+\;C\;h$$

(4) $$n\left(\frac{\text{adenovirus}}{{cm}^{2}}\right)=10^{-2.21}\;C^{0.8}\;+\;C\;h$$

(5) $$n\left(\frac{\text{coxsackievirus}}{{\text{cm}}^{2}}\right)=10^{-2.07}\;C^{0.8}\;+\;C\;h$$

## DISCUSSION

Virus transfer between liquid and skin drives indirect infection risks from interactions with contaminated drinking and recreational water. Here, we quantified virus transfer from liquid to skin for two enteric pathogens, adenovirus type 2 and coxsackievirus B5. Additionally, we compared these transfers with that of a commonly used surrogate, bacteriophage MS2. We demonstrate that the adsorption of the pathogens to the skin was less than the adsorption of MS2, suggesting that virus adsorption to the skin is influenced by the viral species. The finding highlights the need to utilize the pathogen of interest in studies of pathogen-skin interactions, rather than surrogates, when possible.

In a comparison of the results of the present study with the results from a study by Pitol et al. (34), we observed close to a 1-log_10_ difference in the unadsorbed fraction of viruses transferred to the skin for all concentrations tested. The difference observed in the number of unadsorbed viruses on the skin between the two studies is attributed to the experimental method (finger immersion versus liquid droplet), which influenced the film thickness and hence the retention of liquid on the skin (Fig. S2). Within the scope of a risk assessment, the volume of liquid retained on the skin is expected to be different from the volumes retained in this study. The volume retained on skin is influenced by the person-skin characteristics (e.g., hydration of the stratum corneum and hairiness), liquid characteristics (e.g., viscosity and density), and liquid-skin contact activity (e.g., hand immersion in liquid and hand contact with wet cloth) (53). For example, the US EPA Exposure factors handbook (7) provides estimates for water film thickness retained on the skin after different activities, and these estimates vary from 1.32 × 10^−3^ to 4.99 × 10^−3^ cm, depending on the activity performed (7), which is comparable with the estimated film thicknesses measured in the present (1.4 × 10^−3^ cm) and previous (1.1 × 10^−4^ cm) studies.

The amount of bacteriophage MS2 adsorbed per surface area onto the skin was 0.74 log_10_ (5.5 times) greater than that of adenovirus and 0.61 log_10_ (4.1 times) greater than that of coxsackievirus. Therefore, using data from bacteriophage MS2 to estimate the transfer of enteric viruses may overestimate virus transfer and the associated risks. Our results differ from previous findings of virus transfer between liquid and skin, in which virus species had no meaningful influence (difference in effect size, <2%, equivalent to <0.05-log_10_ difference) on virus adsorption for bacteriophages Qβ, Φ6, and MS2 (34). Adenovirus, coxsackievirus, and MS2 have different characteristics, such as genome type, particle size, particle shape, and isoelectric point (Table S1). Based on those characteristics, MS2 shares more features with either adenovirus or coxsackievirus than adenovirus and coxsackievirus shared with one another. However, MS2 adsorbed more to the skin than the two enteric viruses did, and there was no significant difference between adenovirus and coxsackievirus. The difference observed in the number of viruses adsorbed to the skin could be due to differences in other viral properties, such as viral survival and aggregation, and/or differences in counting method (double agar layer [DAL] for MS2 and most probable number [MPN] for enteric pathogens) (40, 54, 55).

Consistent with prior work, the present study shows that the adsorption of virus to the skin is influenced by the seeding concentration of virus in the liquid (34). Specifically, the number of viruses from liquid that adsorb onto the skin is describable as a linear model using the log_10_-transformed concentration of viruses in the liquid as the independent variable. Linear models have been previously used to describe virus adsorption to inanimate surfaces (34, 54). In addition to concentration, virus adsorption on the skin was also associated with virus type and cadaver specimen used. The difference observed between different cadaver specimens indicates that interindividual variation in skin surface properties influences virus transfer. Notably, the difference was similar to that observed for MS2 between different healthy human volunteers (Fig. S4). Differences are likely attributed to the variation on skin characteristics, such as skin microbiota (56), skin humidity, pH, and sebum content (57).

In this study, we expected that when controlling for concentration of virus in seeding liquid, the numbers of unadsorbed viruses on the skin per surface area would be similar for the three viruses tested. This is because the number of unadsorbed viruses per surface area should equal the volume of liquid retained on the skin per surface area times the seeding concentration of virus in the liquid. However, the number of unadsorbed viruses was ∼0.4 log_10_ higher for MS2 than for enteric viruses. The cause of the difference is unclear but may be due to the counting methods used (DAL for MS2 and MPN for enteric pathogens), or variation in virus persistence and/or aggregation. Experiments using the MPN method to quantify MS2 suggest that the counting method (MPN or DAL) influences the variability of the observed data and the number of bacteriophages MS2 enumerated, with larger deviances between the two methods observed at lower concentrations (Fig. S5). Nevertheless, the difference observed in the unadsorbed fraction of viruses on the skin was lower (∼0.2 log_10_) than that observed with the adsorbed fraction (Fig. S3 and Table S2), suggesting the observed difference in the adsorbed fraction (∼0.7 log_10_) is at least partially attributed to virus characteristics.

Our work has some limitations. Only two pathogenic viruses (adenovirus and coxsackievirus) were tested; the transfer of other important viral pathogens (i.e., rotavirus and norovirus) may be different. Similarly, virus retention may vary between strains within the same virus family. Further studies may consider examining the transfer of other viruses to understand the impact of strain type on transfer and/or quantify transfer for other important pathogens. The study also did not quantify the proportion of viruses seeded onto the skin that became irreversibly adsorbed or the proportion of viruses lost due to sample processing (i.e., adsorption to collection tubes). Accounting for either of these proportions was not possible given the order of magnitude difference in the concentration of virus seeded on the skin compared to the concentration of the virus recovered, as well as the inherent variability of the plaque assay. Additionally, virus inactivation during the experiment was not tested herein. However, inactivation rates for enteric viruses, including adenovirus and coxsackievirus, in the environment are relatively low (58). Specifically, inactivation rates for infective MS2, adenovirus, and coxsackievirus in different liquids (i.e., tap water and groundwater) vary from 0.02- to 0.0008-log_10_ reduction per h (59–61). The experiments were carried out within 10 h, so differences in liquid inactivation would have minimal influence on estimates of virus transfer. Differential aggregation of viruses may also influence virus transfer but was not assessed here. Future experiments may consider the impacts of virus persistence and/or aggregation on virus transfer, as these factors are known to be influenced by environmental conditions and virus species and so may also influence transfer estimates (55, 62, 63).

The use of cadaver specimens was shown to offer an alternative testing system to human volunteers for the study of pathogenic viruses. The overwhelming majority of studies on virus and bacteria transferred to the skin use nonpathogenic surrogates in human volunteers (15, 18–20, 34, 64–67). In the present study, we demonstrated that the virus transfer to cadaver skin is similar to the transfer to human volunteer skin. In contrast, MS2 adsorption to synthetic skin (Vitro-Skin) was significantly lower than adsorption to both cadaver and volunteer skin, indicating that synthetic skin is not an appropriate surrogate for studies of virus transfer. The lower complexity of synthetic skin than that of human skin may explain the lower adsorption of bacteriophages onto Vitro-Skin. The outermost layer of the skin, the stratum corneum, is composed of a complex lipid mixture of free fatty acids, ceramides, and cholesterol (68) and is colonized by a highly diverse microbial community (69, 70). Vitro-Skin was not specifically designed to mimic the entire complexity of human skin and its microbiota. Additionally, Vitro-Skin was completely free of dust and organic particles. The particles and the microbiota populating may offer more binding sites for the bacteriophage to attach as they increase the surface area. Further studies are needed to understand the skin characteristics (i.e., surface roughness, microbial community, and lipid content) that are most influential in virus adsorption.

The findings of this study inform microbial risk assessments of water-related activities. To date, risk assessments quantifying the transmission of pathogens calculate the ingested dose using (i) broad assumptions, such as 1 to 10 ml of liquid being accidentally ingested per h while performing different activities (71–74), (ii) self-reported ingestion data obtained from adults performing recreational water activities (30, 75, 76), or (iii) estimates of the thickness of liquid transferred to the skin after contact (31, 33). Estimates reported in this study inform risk assessments of water-related activities that involve indirect transmission of viruses. Using the amount of pathogenic virus transferred from liquid to hand and from hand to mouth describes more mechanistically indirect transmission of viruses than using a liquid ingestion rate (e.g., 1 ml of contaminated liquid ingested/h).

To account for liquid-to-hand transfer in quantitative microbial risk assessment (QMRA) studies of water-related activities, both the number of viruses adsorbed on the skin and the number of viruses in the liquid retained on the skin should be estimated. The results from this study suggest that the number of enteric viruses transferred to the skin per surface area should be modeled as *n* = 10^−1.51 + *bvirus*^ *C*^0.8^ + *C h*, which accounts for viruses adsorbed plus viruses unadsorbed.

## MATERIALS AND METHODS

### Virus propagation, purification, and enumeration.

Bacteriophage MS2 was selected as a pathogenic virus surrogate because of its extensive use as a surrogate for enteric viruses in virus adsorption studies and in transfer studies involving human volunteers (19, 20, 77–82). To grow bacteriophage MS2 (DSMZ 13767), 1 liter of tryptic soy broth (BD Difco, Sparks, MD, USA) containing log-phase Escherichia coli (ATCC 700891 or DSMZ 5695) was inoculated with 10^9^ PFU/ml MS2. After overnight incubation, the medium was clarified by centrifugation for 20 min at 4,000 × *g*, followed by filtration of the supernatant using a 0.2-μm-pore-size syringe filter (VWR International, USA). Finally, the filtered supernatant was concentrated using an Amicon Ultra centrifugal filter device (100 kDa; Merck Millipore).

The double agar layer (DAL) procedure was used to enumerate infective MS2 bacteriophage (34). Briefly, 100 μl of the sample was combined with E. coli in tryptic soy broth (BD Difco, Sparks, MD, USA) with 0.7% agar (BD Difco) and poured into a plate containing 1.5% tryptone soya agar. Positive (10^6^ PFU/ml MS2) and negative (E. coli host with no bacteriophage) controls were included in each experiment.

Human adenovirus type 2 and human coxsackievirus B5 viruses were selected due to their prevalence in water and feces (83–87), relevance for human health (83, 88–91), and the availability of a culture-based quantification method (92). Adenocarcinoma alveolar basal epithelial (A549) cells and buffalo green monkey kidney (BGMK) cells, obtained from the Cell Culture Facility of the University of California Berkeley, were used to propagate the human adenovirus type 2 and coxsackievirus B5, respectively, as previously described (92). Briefly, a flask containing 95% confluent cell monolayer was inoculated with 100 μl of adenovirus or coxsackievirus stock at a concentration of ∼10^6^ most probable number (MPN)/ml. The culture was incubated at 37°C in 5% CO_2_ and 95% humidity until cytopathic effects were observed, which occurred 7 to 10 days after inoculation. The sample was then clarified by centrifugation for 20 min at 4,000 × *g*. Finally, the supernatant was filtered using a 0.2-μm-pore-size filter (VWR International, USA) and concentrated using an Amicon Ultra centrifugal filter device (100 kDa; Merck Millipore). The medium used for culturing the cell lines was Dulbecco's modified Eagle medium (DMEM; Gibco, NY, USA) for A549 cells and minimum essential medium (MEM; Gibco) for BGMK cells. Both culture media were supplemented with 10% fetal bovine serum (FBS; Gibco) and 1% antibiotics (10,000 U/ml penicillin-streptomycin; Gibco) for cell culture and 2% FBS and 1% antibiotics for viral infection.

The MPN method was used to enumerate infective viruses. Briefly, the samples were diluted and spiked into 96-well plates with a 95% confluent cell monolayer in quintuplicate. After virus inoculation, the plates were incubated at 37°C and 5% CO_2_ for 10 to 14 days before counting cytopathogenic units. Every 96-well plate had five wells as a negative control, consisting of a 95% confluent cell monolayer in medium, without virus.

### Study design.

We performed two sets of experiments, studies A and B (Table 1). The objective of study A was to determine if cadaver skin and synthetic skin (Vitro-Skin; Portland, ME, USA) were appropriate human skin surrogates for virus transfer experiments. The objectives of study B were to estimate the transfer of adenovirus and coxsackievirus from liquid to skin and to compare the transfer of both pathogenic viruses with bacteriophage MS2. The results obtained were also compared with the results published by Pitol et al. (34), in which the MS2 transfer from liquid to skin was quantified using a different experimental method (Table 1).

### Virus transfer experiments.

Virus transfer studies were performed on synthetic skin, the arms and hands of volunteers, and the arms and hands of cadavers (Table 1) using an adaptation of a previously published method to quantify virus transfer from liquid to skin (34). After liquid-skin contact, a fraction of viruses remains on the skin due to both virus adsorption to the skin and incomplete removal of virus-containing liquid. Viruses suspended within the liquid remaining on the skin are referred to as unadsorbed viruses, whereas viruses remaining on the skin due to adsorption are referred to as adsorbed viruses (34). Therefore, we defined virus transfer from liquid to skin as the sum of the adsorbed viruses and unadsorbed viruses on the skin after contact. The number of viruses transferred is dependent on the surface area of the contact and the concentration of viruses in the liquid (34). Unadsorbed viruses were recovered using phosphate-buffered saline (PBS [pH 7.4]; Gibco), the same solution used to apply the viruses. A beef extract solution (3% [wt/vol] beef extract [Sigma-Aldrich], 0.1 M glycine [Fluka] [pH 8]), commonly used to desorb virus from surfaces (54, 93), was used to recover the viruses adsorbed on the skin.

A schematic of the method used to quantify virus transfer is shown in the supplemental material (Fig. S1). Briefly, a circular area (5 mm diameter) was delimited on the skin by gently pressing the rim of a 20-μl pipette tip dipped in Vaseline (Vifor Pharma, Switzerland). Subsequently, a 20-μl droplet of PBS containing either bacteriophage (MS2) or human virus (coxsackievirus or adenovirus) was added to the area inside the Vaseline circle. Five seconds after addition, the droplet was removed using a pipette. Longer contact times between inoculated liquid and human skin (up to 30 min) did not influence the extent of bacteriophage transfer to skin (34). After the transfer event, the area inside the Vaseline was sampled by pipetting up and down once using 20 μl of PBS to recover the unadsorbed viruses, followed by pipetting up and down five times using beef extract to recover the adsorbed viruses. To reduce variation in the force applied to recover the adsorbed viruses, all the transfer events were performed by the same researcher. Each experiment had a negative control which consisted of sampling the area inside the Vaseline where no virus was applied.

### Skin preparation. (i) Human volunteer hands and arms.

Volunteers' hands and arms were inspected to ensure the absence of skin damage. Volunteers were asked not to wash their hands or apply any disinfectant before the experiment, as this has been shown to significantly reduce the transfer of viruses (20, 21) and does not mimic typical skin conditions. After the transfer experiments, the hands and arms of the volunteers were disinfected using 70% ethanol (Fisher Scientific), followed by hand washing with soap.

### (ii) Human cadaver hands and arms.

Human cadaver experiments were conducted at the Stanford University BioSkills Laboratory, where the cadaver specimens were provided. Previous studies have suggested that the integrity of cadaver skin is compromised after freezing at −20°C (94). Therefore, all cadavers used were preserved at 4°C without chemical treatment for a period of no longer than 3 weeks prior to the experiments. On the day of the experiment, a specimen (hand with arm) was removed from the body by a BioSkills laboratory technician. Two hours before the experiment, the hand and arm were placed at room temperature. After the transfer experiment was performed, the tested area of the specimen was disinfected using 1% sodium hypochlorite for 15 min and was either stored for use a second time or discarded. If a specimen was used more than once, a new area of the hand or arm was used for subsequent experiments. No specimen was used more than twice. In total, 5 specimens were used for 8 experiments.

### (iii) Synthetic skin.

Vitro-Skin N-19 (IMS, Inc.), a synthetic substrate designed to mimic the surface properties of human skin, was cut in 10-cm^2^ pieces. Hydration of the substrate was carried out according to the manufacturer's instruction. Briefly, a solution of 15% glycerin (Sigma-Aldrich) was placed in the bottom of a closed chamber to regulate the humidity inside the chamber. A sample of Vitro-Skin was placed on a tray inside the humidity-controlled chamber for 16 to 24 h before the experiment. To minimize substrate dryness, the transfer experiments were performed immediately after taking the substrate out of the hydration chamber (<20 min). Each sample was only used once before being discarded.

### Ethical and biosafety approval.

The protocol for the virus transfer studies performed in cadaver specimens was approved by the Stanford University Administrative Panel on Biosafety (APB). The protocol used in the human subject studies was approved by the research ethics committee of ETH Zurich. Written consent from the participants of the human subject studies was obtained before the experiment.

### Statistical analyses.

All statistical analyses were performed using the R statistical software (the R Foundation for Statistical Computing Platform, version 3.4.4). Statistical significance was defined at an α value of <0.05.

## Supplementary Material

Supplemental file 1
