# Supplementary material for: Transfer of Enteric Viruses Adenovirus and Coxsackievirus and Bacteriophage MS2 from Liquid to Human Skin
Source: Appl Environ Microbiol. 2018 Oct 30;84(22):e01809-18. doi: 10.1128/AEM.01809-18 (PMC6210118; doi:10.1128/AEM.01809-18)
Supplement: Supplemental file 1 [file zam022188850s1.pdf]

Supplemental Material:
Transfer of enteric viruses (adenovirus and coxsackievirus) and bacteriophage (MS2) from liquid to human skin

Submitted

to

Applied and Environmental Microbiology

ANA K. PITOL[1,2], HEATHER N. BISCHEL[2,3], ALEXANDRIA B. BOEHM[4], TAMAR KOHN[2], TIMOTHY R. JULIAN*[1,5,6]

[1]Eawag, Swiss Federal Institute of Aquatic Science and Technology, Dübendorf, Switzerland

[2]Laboratory of Environmental Chemistry, School of Architecture, Civil, and Environmental Engineering (ENAC), École Polytechnique Fédérale de Lausanne (EPFL), Lausanne, Switzerland

[3]Department of Civil & Environmental Engineering, University of California, Davis, California, USA

[4]Department of Civil and Environmental Engineering, Stanford University, Stanford, California, USA

[5]Swiss Tropical and Public Health Institute, Basel, Switzerland

[6]University of Basel, Basel, Switzerland

* Corresponding author: Timothy R Julian, Email: tim.julian@eawag.ch, Phone:

+41 58 765 5632

S4. Most Probable Number (MPN) vs Plaque Forming Units (PFU)

Method:

To understand the influence of the counting method –Most Probable Number (MPN) and Double Agar Layer (DAL) method– on the number of bacteriophages enumerated we developed a MPN method to count bacteriophage MS2 by performing the DAL into 96-well plates. Briefly, we aliquoted 200ul of 1.5% Tryptone Soya Agar into each one of the 96 wells and refrigerated the plate at 4°C until used. The day of the experiment we combined visibly turbid *E. coli* in 0.7% Tryptone Soya Agar and poured it into the 96- well plate containing 1.5% Tryptone Soya Agar. We let it dry for 5 min and subsequently we added 2ul of the each one of the serial dilutions of the bacteriophage sample in quintuplicate.

Results:

There was no significant difference in the number of bacteriophage counted using MPN or DAL method for the highest concentration tested, 10^{10} virus/cm^3, (t-test, $t(3)=-0.28$, $p=0.80$). On the contrary, for concentrations of 10^8 and 10^7 virus/cm^3, the number of bacteriophage counted was significantly higher when the method used was PFU as compared with MPN ($t(6)=-13.3$, $p<0.001$), and ($t(5)=-5.1$, $p=0.004$) respectively (Figure S4). The t-test were conducted using Bonferroni adjusted alpha levels of 0.017 per test (.05/3).

Table S1. Characteristics of the bacteriophage and pathogenic viruses tested

Virus	Structure	Shape	Genome type	Genome Size (Kb)	Particle size (nm)	pI	Reference
Bacteriophage MS2	Non-enveloped	Ichosahedral	ssRNA	3.5	26	3.9	(1,2)
Human adenovirus type 2	Non-enveloped	Ichosahedral	dsDNA	35-36	90	3.5- 4	(1,3)
Human coxsackievirus B5	Non-enveloped	Spherical	ssRNA	7.4	30	4.75<br>6.75	(1,4)

Table S2. Multiple regression analysis for the $\log_{10}$-transformed number of viruses unadsorbed number of viruses present in the liquid retained on the skin as a function of the $\log_{10}$-transformed concentration of virus in the liquid, virus type, specimen used, and body part. The table shows the coefficient of the regression (***b***), standard error (***SE B***), standardized regression coefficient (***β***), goodness-of-fit (***R²***), and significance level (**p**). *Reference* refers to the reference group used in the multiple regression analysis.

	Variable	R^2	*b*	*SE B*	*β*	p
**Model 1**		0.69				
	**Intercept**		-2.27			<.001
	**Concentration**		0.93	0.07	0.8	<.001
	**Virus**					
	MS2		*Reference*			
	adenovirus		-0.74	0.09	-0.77	<.001
	coxsackievirus		-0.61	0.09	-1.06	<.001
	**Specimen**					
	1		*Reference*			
	2		-0.09	0.11	-0.05	.448
	3		-0.43	0.16	-0.37	.007
	4		-0.06	0.13	-0.06	.680
	5		-0.17	0.14	-0.3	.202
	**Body part**					
	Arm		*Reference*			
	Hand		0.01	0.09	-0.01	.896
**Model 2**		0.68				
	**Intercept**		-1.51	0.40		<.001
	**Concentration**		0.80	0.06	0.69	<.001
	**Virus**					
	MS2		*Reference*			
	adenovirus		-0.71	0.09	-0.75	<.001
	coxsackievirus		-0.57	0.09	-0.49	<.001

Table S3. Multiple regression analysis for the $\log_{10}$-transformed number of viruses adsorbed on the skin per surface area as a function of the $\log_{10}$-transformed concentration of virus in the liquid, virus type, specimen

used, and body part. The data in this table presents a multiple regression model based on the same data described in Table 2, with an alternative reference virus (adenovirus here compared to MS2 in Table 2). The alternative reference of adenovirus provides insight into the statistical significance of the difference in transfer between adenovirus and coxsackie, which is otherwise unclear form Table 2. Additionally, the reference specimen was changed to specimen 3 from specimen 1 to observe statistical significance of differences between specimen 3 and the other specimens. The table shows the coefficient of the regression (***b***), standard error (***SE B***), standardized regression coefficient (***β***), goodness-of-fit (***R^2***), and significance level (**p**). *Reference* refers to the reference group used in the multiple regression analysis.

	**Variable**	***R^2***	***b***	***SE B***	***β***	**p**
**Model 1**		0.69				
	**Intercept**		3.43248	0.57177		<.001
	**Concentration**		0.93	0.07	0.8	<.001
	**Virus**					
	adenovirus		*Reference*			
	MS2		-0.74	0.09	0.76	<.001
	coxsackievirus		0.12	0.09	0.20	0.181
	**Specimen**					
	3		*Reference*			
	1		0.43	0.16	0.27	0.007
	2		0.34	0.13	0.30	0.010
	4		0.37	0.14	0.38	0.011
	5		0.25	0.15	0.42	0.102
	**Body part**					
	Arm		*Reference*			
	Hand		0.01	0.09	-0.01	0.896

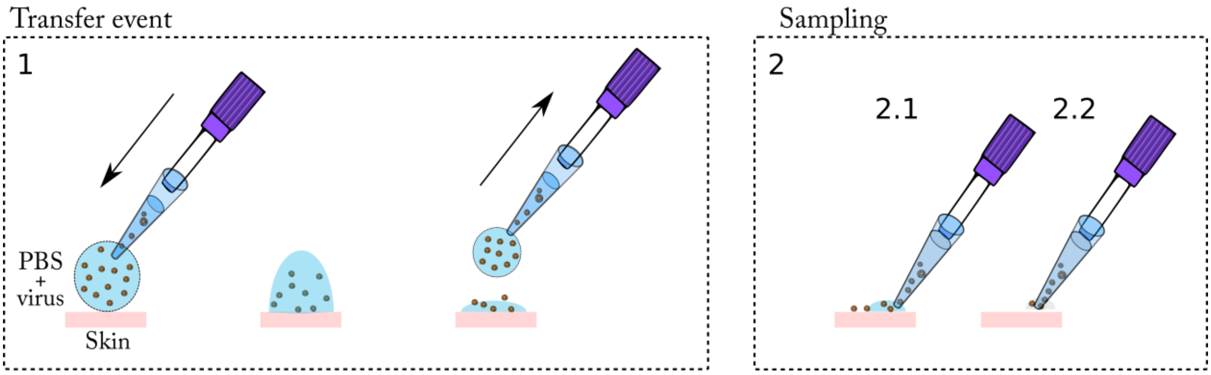

Figure S1. Virus transfer method. 1) Twenty microliters of PBS containing viruses (MS2, adenovirus, or coxsackievirus) at a concentration of 10^6-10^8 Plaque Forming Units (PFU) or Most Probable Number (MPN)/ml were added to a circular area in the skin ($0.19 cm^2$) delimited using Vaseline. The inoculum was left on the skin for five seconds (transfer event) and was subsequently removed from the skin. **2)** Subsequently, the area inside the Vaseline was sampled by **2.1)** pipetting up and down once using PBS to remove the "unadsorbed" viruses, followed by **2.2)** pipetting up and down 5 times using beef extract solution to remove the viruses adsorbed on the skin. Figure adapted from Pitol et al. (2017)[5].

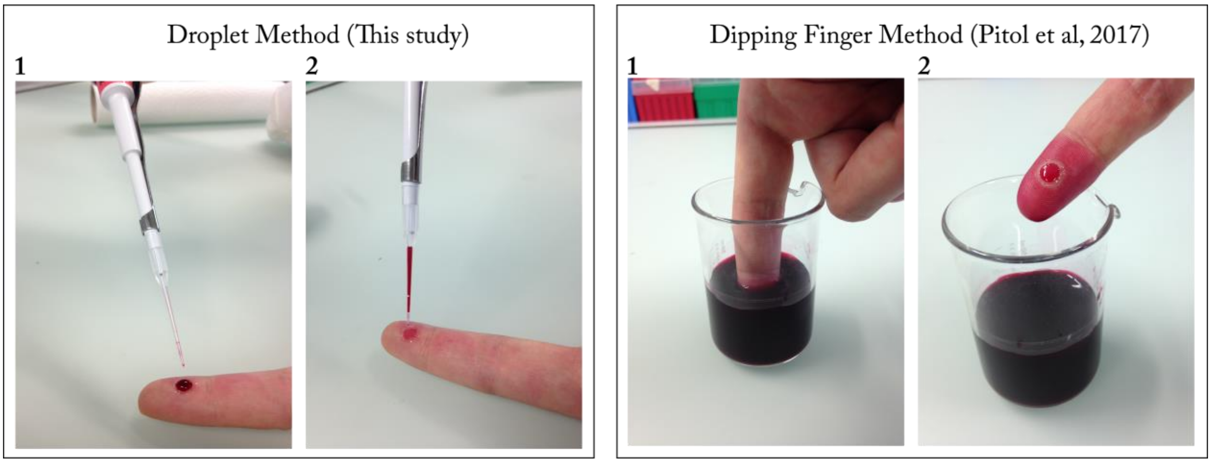

Figure S2. Different transfer methods. Illustration of the transfer method used in this study (droplet method) as compared with the method used by Pitol et al. (2017) [5] (finger dipping method). To illustrate the differences between the two methods we performed a simulation using beet juice. Beet juice was selected due to its color only with illustration proposes. The transfer method in this study consisted of applying and removing a droplet of contaminated liquid on the skin using a pipette. In contrast, the transfer method of Pitol et al. 2017[5] consisted of dipping the finger directly into the liquid.

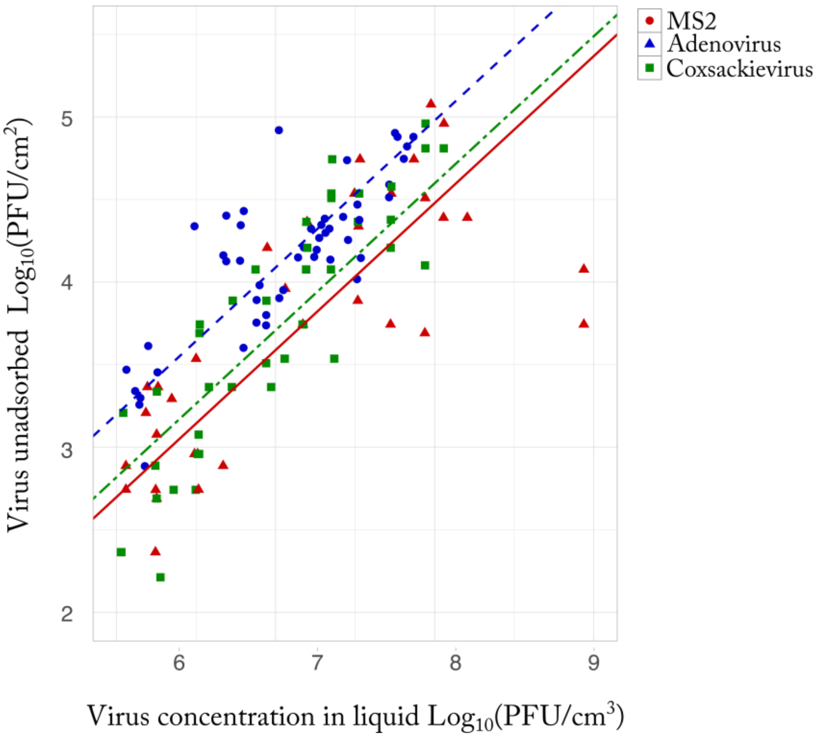

Figure S3. Number of bacteriophages and pathogenic viruses retained in the residual liquid on the skin (unadsorbed fraction) as a function of seeding concentration. The plots show the $\log_{10}$-transformed unadsorbed MS2 (red circles), adenovirus (blue triangles), and coxsackievirus (green squares) surface density on the skin as a function of the $\log_{10}$-transformed concentration of virus in the liquid. The regression lines represent the multiple regression model (Model 2, Table S2) for the number of viruses adsorbed per surface area as a function of concentration.

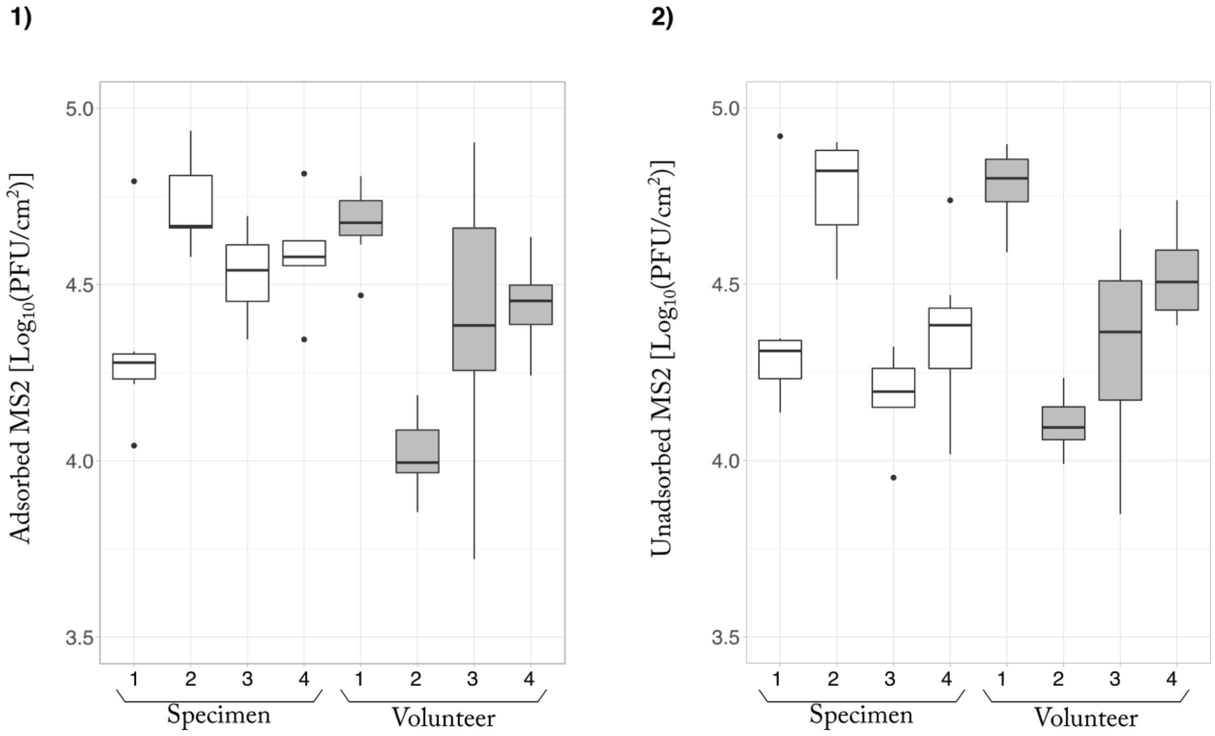

Figure S4. Variation in the log_{10}-transformed number of bacteriophage MS2 transferred –adsorbed and unadsorbed– to the skin for volunteers and cadaver specimens. The box plots summarize the number of MS2 bacteriophages per surface area that area **1)** adsorbed and **2)** unadsorbed on cadaver specimens and volunteers hands and/or arms. The transfer studies were conducted using a seeding concentration of bacteriophage MS2 of $10^{7.5}$ PFU/ml. The box plots summarize data of 7 individual transfers for specimen/volunteer, the bottom and top of the box plots represent the 25th and 75th percentiles, the line in the center represents the median value and the whiskers extend to the highest and lowest values. Individual dots represent outliers, defined as values greater than 1.5 interquartile range (box lengths).

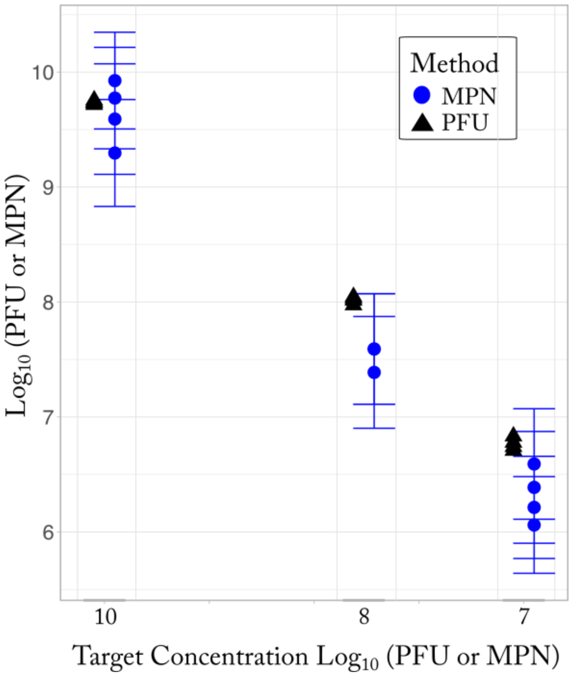

Figure S5. Influence of counting method on MS2 quantification. The plot shows the number of MS2 as a function of counting method (Plaque forming units (PFU) = black triangles; Most probable number (MPN) = blue circles) for three experiments performed at a concentration of ~10^{10}, 10^{8}, 10^{7} PFU/ml respectively. Each experiment had 4 replicates. The MPN data is presented together with its 95% CI.

REFERENCES

1. ViralZone: a knowledge resource to understand virus diversity. Hulo C, de Castro E, Masson P, Bougueleret L, Bairoch A, Xenarios I, Le Mercier P. Nucleic Acids Res. 2011 Jan;39:D576-82. Retrived from http://viralyone.expasi.org
2. Armanious A, Aeppli M, Jacak R, Refardt D, Sigstam T, Kohn T, Sander M. 2015. Viruses at solid-water interfaces: A systematic assessment of interactions driving adsorption. Environ Sci Technol 2:732–743.
3. Wong K, Mukherjee B, Kahler AM, Zepp R, Molina M. 2012. Influence of inorganic ions on aggregation and adsorption behaviors of human adenovirus. Environ Sci Technol 46:11145–11153.
4. Michen B, Graule T. 2010. Isoelectric points of viruses. J Appl Microbiol 109:388–397.
5. Pitol AK, Bischel HN, Kohn T, Julian TR. 2017. Virus Transfer at the Skin-Liquid 593 Interface. Environ Sci Technol 51:14417–14425
